# Immunotherapy for Renal Cell Carcinoma

**DOI:** 10.1155/2010/284581

**Published:** 2011-01-03

**Authors:** Momoe Itsumi, Katsunori Tatsugami

**Affiliations:** Department of Urology, Graduate School of Medical Sciences, Kyushu University, 3-1-1 Maidashi, Higashi-ku, Fukuoka 812-8582, Japan

## Abstract

Immunotherapy plays a significant role in the management of renal cell carcinoma (RCC) patients with metastatic disease because RCC is highly resistant to both chemotherapy and radiation therapy. Many reports illustrate various approaches to the treatment of RCC, such as cytokine-, antigen- or dendritic cell- (DC-) based immunotherapy, and the safety and effectiveness of immunotherapy have been highlighted by multiple clinical trials. Although antitumor immune responses and clinically significant outcomes have been achieved in these trials, the response rate is still low, and very few patients show long-term clinical improvement. Recently, the importance of immune regulation by antigen-presenting cells (APC) and regulatory T cells (Treg cells) has also been discussed. The authors outline the principles of cell-mediated tumor immunotherapy and discuss clinical trials of immunotherapy for RCC.

## 1. Introduction

Renal cell carcinoma (RCC), a glandular carcinoma, accounts for approximately 85%–95% of adult malignant kidney cancer cases [1]. Patients with advanced or metastatic disease have a poor prognosis, with a 5-year survival rate of less than 15%. Surgical treatment is effective, even in patients with advanced or metastatic RCC, because of its high resistance to chemotherapy and radiation therapy. Immunotherapy using interferon (IFN)-*α* and/or interleukin (IL)-2 has shown promising anti-tumor activity in RCC [2–4]. However, these cytokines have a positive effect in only 10%–20% of cases [5]. Like melanoma, RCC is classed as an immunogenic tumor based on its response rate to immunotherapy, the incidence of spontaneous regression, and the high level of tumor T cell infiltration. Despite its immunogenicity, only a few CD8^+^ cytotoxic-T-lymphocytes (CTLs), which can efficiently eliminate RCC cells, have been isolated [6]. This is in line with the small number of RCC-associated antigens that have so far been identified, thereby limiting the trials of candidate vaccines in these patients [7, 8]. 

Recently, tumor immunotherapy using DC has been shown to have therapeutic potential for malignant tumors. Moreover, nonmyeloablative stem cell transplantation (NST), which was developed for the treatment of leukemia, is effective against RCC [9, 10] and other solid tumors [11]. In this review, we discuss the current status of cell-mediated tumor-specific and nonspecific immunotherapy for RCC.

## 2. Tumor-Specific and Non-Specific Immunotherapy

*In vivo* studies show that cellular immunity mediated by T cells, natural killer (NK) cells or NK T cells plays a central role in the eradication of tumors. Since 1980, many attempts have been made to administer anti-tumor cells to cancer patients. In the late 1980s, human tumor antigens were identified and tumor-specific cellular immunity mediated via these tumor antigens received a lot of attention. Also, the administration of cytokines that activate cellular anti-tumor responses, including those mediated by T cells and NK cells, has been the subject of much research. It is thought that IFN-*α* induces Th1 cytokine production, thus promoting anti-tumor activity by cells that elicit cytotoxicity by acting directly on the tumor [12]. 

IL-2 is a growth/differentiation factor for NK cells and T cells, which induces and maintains the cytotoxicity, both these cell types [13]. Because cytokine treatment induces nonspecific anti-tumor activity, it is known as nonspecific immunotherapy.

In 1984, Mule et al. reported lymphokine-activated killer (LAK) cell treatment of tumors using inducible cultured cells [14]. Culturing immune cells isolated from a cancer patient's peripheral blood, or excised tumor tissue, with IL-2 causes them to differentiate into LAK cells. Since the second half of the 1980s, treatment using LAK cells has been attempted in several facilities [15, 16]. However, because the treatment method causes severe side effects, it was never established as an effective treatment method. LAK cells have no tumor specificity because they are induced in culture in response to IL-2 alone and not by tumor antigens. Thus, it was thought that the adoptive transfer of LAK cells might result in damage to normal host cells *in vivo*.

Since Van Der Bruggen et al. identified tumor antigens that were specifically recognized by T cells in a melanoma-bearing patient [17], research became more focused on tumor-specific immunotherapy. Though LAK cells, CTLs, macrophages, NK cells and NKT cells are all involved in host immune response against tumors, CTLs are now thought to be one of the most important factors responsible for anti-tumor immunity.

## 3. Immunotherapy Using Inactivated Tumor Cells and Gene Modified Tumor Vaccines (GMTV)

Immunotherapy using inactivated tumor cells or tumor lysates is based on the idea that tumor cells express antigens that induce anti-tumor immune responses [18–22] (Table 1). Because immunotherapy using tumor cells is relatively straightforward, Jocham et al. undertook a large-scale randomized controlled trial and reported that the “nonreplaced phase” after surgery for kidney cancer was extended by an autologous tumor vaccine [20]. The percentage of vaccinated patients showing no disease progression 5 years after treatment was 77.4% compared with 67.8% of the controls.

Both cytokines and antigen-presenting cells are important for the induction of effective immune responses [23]. Thus, GMTV was used to introduce virus-expressing cytokines, or costimulatory molecules, into tumor cells (Table 1) [18–22, 24–33]. GMTV-immunotherapy introducing cytokine transgene, such as GM-CSF or IL-2, or costimulatory molecule transgene such as B7-1 into autologous irradiated tumors, has been carried out. However, these studies were disappointing in terms of a significant clinical response, such as tumor regression. Though the use of multiple tumor antigens should induce a greater immune response, one cannot rule out the possibility of unintentionally inhibiting anti-tumor immunity or of eliciting nonspecific immune responses.

## 4. Peptide-Based Immunotherapy

Since the development of the SEREX method, which enables the identification of tumor antigens from cDNA libraries, many peptide-based vaccination studies have been undertaken. Because the effective induction of anti-tumor immunity using single peptides is difficult, MHC class II peptides have been used along with adjuvants (Table 2) [34–40]. HSPPC-96 (vitespen) is a heat shock protein. It is a peptide complex, in which the heat shock protein plays the role of an adjuvant. However, a recent randomized phase III study suggested that this complex did not improve recurrence-free survival rates [41]. Further studies are required to see whether antigen-specific T cells homogeneously induced by a single tumor antigen can be effective against a diverse population of tumor cells.

## 5. DC-Based Immunotherapy

**Figure 1:**
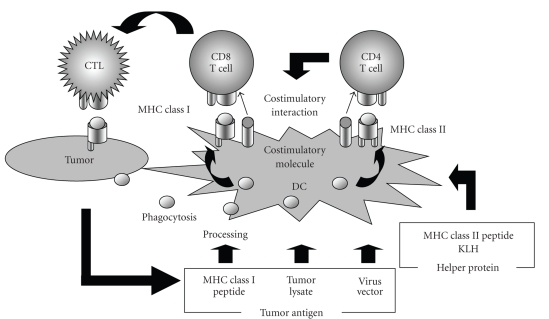
CTL induction by Apcs. Antigens are taken up and degraded into peptide fragments by antigen presenting cells (APC), such as immature DC. At some point on their path to the cell surface, newly synthesized MHC class II or I molecules bind the peptide antigen fragments and transport the peptides to the cell surface. CD8^+^ T cells recognizing the antigen expressed by weakly costimulatory cells become activated only in the presence of CD4^+^ T cells bound to the same APC. This happens via CD4^+^ T cells recognizing antigens presented by APCs and being triggered to induce increased levels of costimulatory activity by the antigen-presenting cell. The CD4^+^ T cells also produce increased amounts of IL-2, which drives CD8^+^ T cell proliferation. CD8^+^ T cells then become cytotoxic T lymphocytes (CTL).

Antigens processed within the proteasome of tumor cells are presented on major histocompatibility antigen (MHC) class I molecules of tumor cell as tumor antigen peptides that CTLs recognize, thus triggering CTL-mediated cytotoxicity. However, CTLs are not activated by direct recognition of the antigens expressed by tumor cells; they need help from dendritic cells (DCs) and CD4^+^ helper T cells. To activate a CD8^+^ T cell to become a CTL, engagement of the T cell receptor with a peptide antigen presented by an MHC class I molecule is not enough. The T cell must also recognize a costimulatory molecule (e.g. CD80 or CD86) (Figure 1). Moreover, antigen presenting cells (APCs) are activated through their interaction with CD4^+^ T cells, and then they express various costimulatory molecules. DCs are the most well-known and efficient APCs and are present in various tissues, including lymphoid and nonlymphoid organs and the blood, where they take up both particulate and soluble antigens before migrating to the lymph nodes to induce immune responses. Subsequently, DCs present antigen to T cells in the lymph nodes and induce antigen-specific immune responses, including the induction of CTLs. DCs also present antigen to other cells, including NK cells.

Clinical trials of DC therapy are listed in Table 3 [23, 35, 36, 42–59]. Although immunotherapy using DCs and nonautologous tumor cells seems to induce host immune cells to recognize tumor cells, there is still the possibility of alloreactive immune responses induced by nonself-antigens. Because nonautologous DCs (allo-DCs) may be attacked by the host immune system, immunotherapy using autologous-DCs (auto DCs) might be more effective *in vivo*. To date, all reports regarding DC treatment are of phase I/II trials incorporating different methodologies. Although delayed-type hypersensitivity reactions in response to tumor cell lysates or keyhole limpet hemocyanin (KLH) and the production of IFN-*γ* by antigen-specific lymphocytes were observed, the number of patients showing a positive clinical response was still low. 

We also used IFN-*α* as an adjunctive agent for DC therapy. As previously noted, IFN-*α* induced an environment conducive to DC activation and enhanced migratory competence [60, 61]. We evaluated the efficacy of DC-therapy in combination with IFN-*α* in patients with advanced RCC. After 4 months of vaccinations, five patients had stable disease and two had progressive disease. In six patients, the time-to-progression was prolonged compared with that seen after previous cytokine treatment. Because cytokine combination therapy induces the proliferation and maintenance of DC-activated T cells, combination therapy using IL-2 is reasonable. However, Oosterwijk et al. reported that combination therapy with IL-2 plus DCs was no more effective than DCs alone [44]. Recently, it was reported that IL-2 participates in the maintenance of regulatory T cells (Tregs), which suppress immune responses [62]. Further study of the role of IL-2 in immunotherapy is required.

## 6. Nonmyeloablative Stem Cell Transplantation (NST)

Though NST was developed for the treatment of leukemia, it began to gain attention as a treatment for solid tumors. In 2000, Childs et al. performed NST on 19 renal carcinoma patients and reported a success rate 53%; three patients were in complete remission and seven patents were in partial remission. Previous reports have highlighted the important role played by cellular anti-tumor immunity, including that mediated by donor T cells in graft versus host disease (GVHD) and the graft versus tumor effect (GVT); the appearance of GVHD induced by transplantation of donor T cells is inversely correlated with the rate of tumor recurrence. Recurrence is especially high in T cell-depleted stem cell transplants, and the administration of donor lymphocytes effectively reduces the incidence of recurrence [63, 64]. Donor T cells induce GVHD/GVT against recipient antigens, including MHC molecules, minor histocompatibility antigens and tumor cell-specific antigens. An effective GVT response can be induced if the antigen distribution between normal cells and tumor cells can be identified, and if donor T cell responses against normal cells can be controlled. Thus, in NST, the mechanism by which tumor specific immunity is induced is very important, and a recent study attempted to address the question of how this response was activated [65].

When the patient receives immunosuppressive treatment for GVHD, it might also cause suppression of the associated anti-tumor effects. In these patients, the differentiation of mononuclear cells into DCs is inhibited *in vitro *[66]. Therefore, when treating a patient with NST, one should bear in mind possible aggravation of the neoplasm by immunosuppressive therapy directed against GVHD.

## 7. Regulatory CD4^+^ T Cells and the Tumor

Recent research shows that CD4^+^ T cells constitutively expressing the IL-2 receptor *α*-chain (CD25) act in a regulatory capacity by suppressing the activation and function of other T cells [67]. Their physiological role is to protect the host against the development of autoimmunity by regulating immune responses against antigens expressed by normal tissues [68, 69]. Since tumor antigens are largely self-antigens, these so-called Treg cells may also prevent the tumor-bearing host from mounting an effective antitumor immune response. Previous studies have shown that elevated numbers of CD4^+^CD25^+^ Tregs can be found in patients with advanced cancer [70] and that high Treg frequencies are associated with reduced survival [71]. In our experiments into cytokine therapy for RCC patients, the number of CD4^+^ and FoxP3^+^Treg cells was significantly decreased after IFN-*α* treatment, and Treg cell levels before treatment correlated with the clinical response [72]. The important role of CD4^+^CD25^+^ Tregs in controlling tumor growth was further highlighted by the demonstration that depletion of Tregs using anti-CD25 antibodies evokes effective antitumor immunity in mice [73, 74]. Dannull et al. used a recombinant IL-2:diphtheria toxin conjugate (DAB_389_IL-2; also known as denileukin diftitox and ONTAK) to eliminate CD25-expressing Tregs in metastatic RCC patients, and reported that depletion of Tregs in RCC patients followed by vaccination with tumor RNA-transfected DCs led to improved stimulation of tumor-specific T cells compared with vaccination alone [75]. It will be critical to collect accurate information regarding Tregs to address the clinical efficacy of such strategies in cancer patients.

## 8. Conclusions

The use of immunotherapy using cultured cells, such as DCs, to treat large numbers of patients, and the conduction of large-scale studies are difficult because of the problems associated with the need for adequate culture facilities and appropriate culture techniques. Because of the complexity of the immune responses involved, it is difficult to evaluate the efficacy of immunotherapy compared with other treatments. However, as it is clear that the immune system plays a significant role in the control of tumors, continued analysis of the mechanisms involved in tumor immunity and the development of new immunotherapies are vital.

## Figures and Tables

**Table 1:** Immunotherapy using inactivated tumor cells and a gene modified tumor vaccine (GMTV).

Authors	Vaccine	Adjuvant	Patients	Duration of PFS/RFS	Results
Galligioni	Auto irrad tumor	BCG	120	13 mo	5-year DFS 63%
					(control 72%) *P* = .21
Schwaab	Auto irrad tumor	BCG, IFN-*α*, IFN-*γ*	14	—	3 MR, 5 SD, l PD
Dillman	Auto irrad tumor	BCG, IFN-*α*, IFN-*β*	25	2.4 mo	median survival 33.4 mo,
		GM-CSF, Cy			5-year survival 43%
Jocham	Auto lysate	None	379	47.8 mo	5-year PFS 77.4%
					(control 67.8%) *P* = .02
Dudek	Auto LMI	None, Cy, Cy+IL-2	31	2.8 mo	None: 5 SD, Cy: 4 SD,
					Cy+ IL-2: 1PR 3 SD
May	Auto lysate	None	495	—	5 year,10 year OS: 80.6, 68.9%
					(control 79.2, 62.1%) *P* = .066
Simons	Auto irrad tumor	None	16	—	1 PR
	+ GM-CSF				
Wittig	Auto irrad tumor	Oligonucleotide**s **	10	—	1 CR, 1 PR, 1 MR, 2 SD, 5 PD
	+ GM-CSF, IL-7				
Antonia	Auto irrad tumor	IL-2	15	—	2 PR, 2 SD
	+ B7.1 gene				
Tani	Auto irrad tumor	None	6	—	1 SD, l MR
	+ GM-CSF				
Pizza	Auto irrad tumor	None	30	170.5 dy	1 CR, 4 PR, 9 SD
	+ IL-2				
Moiseyenko	Auto irrad tumor	None		3 mo	1 SD, l MR
	+ tag7/PGPR-S gene		4		
Fishman	Auto irrad tumor	IL-2	39	—	1 CR, 2 PR, 24 SD
	+ B7.1 gene				
Buchner	Auto irrad tumor	None	12	5.3 mo	PFS 5.3 mo, OS 15.6 mo
	+ B7.1, IL-2 gene				

LMI: large multivalent immunogen, Cy: cyclophosphamide, DFS: disease-free survival, Os: overall survival, PR: partial response, MR: mixed response, SD: stable disease, PD: progressive disease, PFS: progression-free survival, RFS: recurrence-free survival.

**Table 2:** Peptide-based immunotherapy.

Authors	Stage	Vaccine	Adjuvant	Patients	Duration of PFS/RFS	Results
Uemura	mRCC	CA9-derived peptide	Incomplete Freund's adjuvant	23	12.2 mo	3 PR, 6SD
Iiyama	mRCC	WT 1-peptide	Incomplete Freund's adjuvant	3	—	2 SD
Suekane	mRCC	4 different peptides	None, IFN-*α*, IL-2	10	23 wk	6 SD
Wood	cT1b-T4N0M0 or T ant N1-2 M0	HSPPC-96 (vitespen)	None	728	1.9 yr	No difference in recurrence-free survival
Jonasch	mRCC	HSPPC-96 (vitespen)	None	60	65 dy	2 CR, 2 PR, 7 SD

mRCC: metastatic RCC, PADRE: pan-MHC class II binding peptide, Auto mDC: autologous mature DC, CR: complete response, PR: partial response, SD: stable disease, PFS: progression-free survival, RFS: recurrence-free survival.

**Table 3:** DC-based immunotherapy.

Authors	Antigen	DC	Adjuvant	Patients	Duration of PFS/RFS	Results
Oosterwijk-Wakka	Auto lysate	Auto imDC	KLH/IL-2	12	—	8 SD, 4 PD
Marten	Auto lysate	Auto mDC	KLH	15	—	1 PR, 7 SD, 7 PD
Holtl	Auto & Allo lysate	Auto mDC	KLH	27	20.4 mo	2 CR, 1 PR, 7 SD, 17 PD
Azuma	Auto lysate	Auto imDC	KLH	3	—	1 NC, 2 PD
Marten	DC*/*auto tumor fusion	Allo mDC	—	12	—	4 SD, 8 PD
Su	tumor RNA	Auto imDC	—	10	—	not evaluated
Gitliz	Auto lysate	Auto imDC	—	12	—	1 PR, 3 SD, 8 PD
Barbuto	DC*/*auto tumor fusion	Allo mDC	—	19	5.7 mo	30 R, 14 SD, 2 PD
Avigan	DC*/*auto tumor fusion	Auto imDC	KLH	13	4.2 mo	5 SD, 8 PD
Pandha	Allo lysate	Auto imDC	KLH	5	—	2 SD
Arroyo	Auto lysate	Auto mDC	KLH	5	9.6 mo (5–16)	3 SD
Holtl	Auto & Allo TuLy	Allo mDC	KLH/Cy	20	22.3 mo	2 MR, 3 SD, 15 PD
Wierecky	MUC-1 peptide	Auto mDC	PADRE	20	10.8 mo (4–24)	1 CR, 2 MR, 2 PR, 5 SD, 10 PD
Bleumer	CA9 peptide	Auto mDC	KLH CA9 class II peptide	6	—	6 PD
Wei	DC/auto tumor fusion	Auto mDC	IL-2	10	7 mo (5–12)	1 PR, 3 SD, 6 PD
Matsumoto	Auto lysate	Auto mDC	KLH	3	—	1 SD, 2 PD
Kim	Auto lysate	Auto mDC	KLH	9	5.2 mo	1 PR, 5 SD, 3 PD
Berntsen	Lysate or surviving and telomerase peptides	Auto mDC	IL-2	27	2.7 mo	13 SD, 14 PD
Tatsugami	Auto TuLy	Auto mDC	IFN-*α*	7	7.8 mo	5 SD, 2 PD
Zbou	DC/auto tumor fusion	Allo mDC	—	10	—	1 PR, 6 SD, 3 PD

Cy: cyclophosphamide, PADRE: pan-MHC class II binding peptide, Auto mDC: autologous mature DC, Allo imDC: allogeneic immature DC CR: complete response, PR: partial response, MR: mixed response, SD: stable disease, OR: objective response, PD: progressive disease, PFS: progression-free survival, RFS: recurrence-free survival.
